# Habitat disturbance and hydrological parameters determine the body size and reproductive strategy of alluvial ground beetles

**DOI:** 10.3897/zookeys.100.1427

**Published:** 2011-05-20

**Authors:** Michael Gerisch

**Affiliations:** UFZ - Helmholtz Centre for Environmental Research, Department Conservation Biology, Permoserstr. 15, 04318 Leipzig, Germany

**Keywords:** life-history traits, environmental variability, species sorting, trait shifts, floodplain, ecosystem processes

## Abstract

Environmental variability is the main driver for the variation of biological characteristics (life-history traits) of species. Therefore, life-history traits are particularly suited to identify mechanistic linkages between environmental variability and species occurrence and can help in explaining ecological patterns. For ground beetles, few studies directly related species traits to environmental variables. This study aims to analyse how life-history traits of alluvial ground beetles are controlled by environmental factors. I expected that the occurrence of species and the occurrence of specific traits are closely related to hydrological and disturbance parameters. Furthermore I expected most of the trait-variation to be explained by a combination of environmental variables, rather than by their isolated effects. Ground beetles were sampled in the year 2005 in floodplain grassland along the Elbe River in Germany. I used redundancy analysis to quantify the effects of hydrological, sediment, and disturbance related parameters on both species occurrence and species traits. I applied variation partitioning to analyse which environmental compartments explain most of the trait variation. Species occurrence and trait variation were both mainly controlled by hydrological and flood disturbance parameters. I could clearly identify reproductive traits and body size as key traits for floodplain ground beetles to cope with the environmental variability. Furthermore, combinations of hydrological, habitat disturbance, habitat type, and species diversity parameters, rather than their isolated effects, explained large parts of ground beetle trait variation. Thus, a main conclusion of this study is that ground beetle occurrence is mainly determined by complex, multi-scale interactions between environmental variability and their life-history traits.

## Introduction

Observing the occurrence of species and evaluating the response of biodiversity to changing environmental conditions is a major task of ecologists. With increasing ecological knowledge, however, the scientific focus shifted from purely observational to rather explanatory and predictive approaches. Recent attempts try to understand the observed occurrence patterns by focusing on the relationships between environmental variability and the life-history traits of organisms (Naeem and Wright 2003). Life history traits are biological characteristics of species allowing them to survive in their environments, including morphological, behavioural, and physiological characteristics. Current theory, like the habitat templet theory (Townsend et al. 1997), predicts that species traits are mainly constrained by the environmental variability of their habitats and that abiotic factors act like filters, sorting organisms with unique trait combinations appropriate for specific habitat conditions (Statzner et al. 2004). In the past years, several studies successfully applied this theory to determine or predict biodiversity effects of altering environmental conditions and showed the suitability of life-history traits for ecological research. For example, Dalgleish et al. (2010) highlighted the usefulness of trait-based approaches to predict species vulnerability to climate change. Snyder (2008) noted that life-history traits can reveal how species can coexist and several studies described the effects of environmental variables on species traits (e.g. Lehsten et al. 2009; Pausas et al. 2004; Ilg and Castella 2006). The main conclusion of these studies is that functional traits of organisms can explain the ecological response of species (Lavorel et al. 1997). Thus, functional approaches can be seen as an extension of traditional ecological research, as they can reveal general assembly rules to explain ecosystem processes, and to give sound ecological interpretations.

Previously, such analyses were mainly applied to plants, but an increasing number of studies directly related environmental variables also to ground beetle life-history traits. Gobbi and Fontaneto (2008) noted that proportions of short winged, large and predatory species were negatively related to habitat disturbance. Similar results were found by Pizzolotto (2009) and Ribera et al. (2001), stressing that management intensity can influence trait dispersion and morphological characteristics of ground beetles, such as body size or wing morphology. For agricultural landscapes Hendrickx et al. (2009) found that especially ground beetles with low dispersal ability are threatened by habitat fragmentation and Lambeets et al. (2008) demonstrated multiple trait shifts of ground beetles along gradients of flood disturbance. The main conclusion of all these studies is that life-history traits of ground beetles are strongly affected by a variety of different environmental variability in a large range of different habitats.

Analysing trait-environment relationships is especially suitable in naturally dynamic landscapes, because this allows for observing biological patterns without elaborately manipulate environmental conditions (Henle et al. 2006). Floodplains provide exceptional opportunities for such kind of research, since the episodic alternation of floods and droughts causes high spatio-temporal habitat heterogeneity (Tockner and Stanford 2002), being one of the most important drivers for species assemblages and the high species richness of these ecosystems (Adis and Junk 2002). Floodplain faunal species are therefore expected to display a large range of adaptations and strategies to cope with varying environmental conditions (Robinson et al. 2002). However, given this high biotic and abiotic variety of floodplains, mechanistic linkages between environmental variability and life-history traits of organisms are difficult to reveal and thus still insufficiently understood. This is to some degree also true for ground beetles, although they are one of the best studied, most species rich and abundant macroinvertebrate taxon in terrestrial and semi-terrestrial habitats and particularly suitable for the investigation of species-environment relationships (Lövei and Sunderland 1996; Rainio and Niemelä 2003). Recently, some considerable progress has been made to identify the life-history traits of ground beetles to understand their response to floodplain dynamics. Most of the species are good flyers, which enables them to actively evade rising floodwaters and to quickly recolonise the habitats after flooding (Desender 1989). Additionally, a huge amount of alluvial ground beetles are habitat generalists (Weigmann and Wohlgemuth-von-Reiche 1999), which may increase the chance of finding surrogate habitats and to quickly recolonise habitats after flooding. The adults of several alluvial species can stay submerged for a considerable time period and are thus able to outlast flood events for a certain time in the floodplain (Siepe 1989; Rothenbuecher and Schaefer 2006). In contrast, ground beetle larvae are rather intolerant to hydrological stress (den Boer and den Boer-Daanje 1990) and therefore many alluvial species develop in less flood exposed habitats (Rothenbuecher and Schaefer 2006). Spring reproduction is another crucial strategy to ensure reproductive success in these highly dynamic floodplain habitats. Early reproduction enables the larvae to develop during summer, which is usually a period of low hydrological disturbance, and thus can decrease larval mortality and increase reproductive success (Thiele 1977).

Despite the general knowledge of ground beetle survival strategies in floodplains, it is yet not clear how environmental variability controls the distribution of particular traits within species assemblages. Lambeets et al. (2008) and Bates et al. (2006) gave some first insight, as they directly related floodplain variables to specific life-history traits of the species. They stressed the importance of flood disturbance and soil conditions on the variation of species traits. However, these studies were conducted on river banks, being characterised by an extremely high disturbance regime with rapidly altering environmental conditions. For other habitats, like less disturbed floodplain grasslands, other parameters might be of greater importance for the species. The primary aim of this study is to explain the occurrence of ground beetles by linking species life-history traits with environmental variability and species occurrence patterns in floodplain grassland. I expect that the occurrence of ground beetle species and the variation of their traits are strongly affected by hydrological and disturbance related parameters. Due to the environmental complexity of floodplain habitats I further hypothesise that most of the trait variation will be explained by a combination of different environmental variables, rather than by their isolated effects.

## Methods

### Study area

The study was conducted at the UNESCO Biosphere Reserve “Elbe River Landscape” in Central Germany at the Elbe River. With a length of about 1,100 km and a catchment area of about 150,000 km2 the Elbe River is the third largest stream in Germany and ranks among the largest streams in Europe. The mean annual discharge of the Elbe River ranges from 336m3/s upstream to 730m3/s downstream. The water level is mainly dominated by snow-melt in spring and erratic precipitation over the year, inducing high discharge in winter and spring, and low discharge in summer. In general, flood regime and floodplain habitats of the Elbe River in Central Germany can be considered close to the natural state (Scholten et al. 2005).

**Figure 1.**
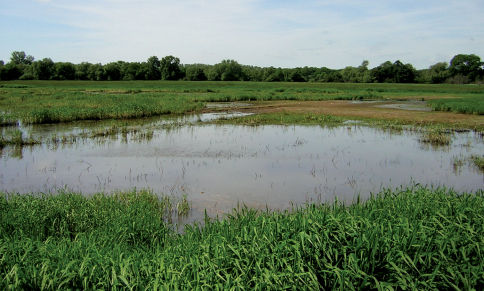
Grassland habitats displaying different hydrological conditions in the study site Steckby. Copyright Mathias Scholz (UFZ, Leipzig).

The survey was carried out in the year 2005 on 36 plots located in seasonally flooded grasslands. The study site is located near the village of Steckby, close to Dessau town in the state of Saxony-Anhalt. The plots were located following a stratified, randomised design. For this, the study site was subdivided into three habitat types regarding vegetation and soil morphology: floodchannels, humid grasslands and mesophilous grasslands. The sampling plots were then randomly located within each of the three habitats (see Henle et al. 2006 for a detailed description of the study design). The study site is characterised by a mosaic of higher and lower areas, which are differently exposed to floods (Fig. 1), whereas the more elevated and dryer areas were cut twice a year and the lower ones (e.g., floodchannels) were spared from utilisation. On each plot five pitfall traps were installed and filled with a 7% solution of acetic acid and a detergent to reduce surface tension. The traps were exposed from May to June and from September to October with a trap exposure time of 28 days per period. All adults were determined to species level and stored in a solution of two-thirds ethanol (70%) and one-third acetic acid (30%).

### Life-history traits

Information on the life-history traits of ground beetles were queried from a self-compiled database. The included trait data came from standard references on Central European ground beetles, mostly determination keys and ground beetle compendia. Altogether 18 traits with 60 trait categories, ranging from biological and morphological to ecological characteristics, were included in the database. For this study I used 8 traits and 25 trait categories to describe the effects of environmental variables on the variation of the traits. See Appendix II for an overview of the traits included in the database and the ones used in this study including the references used to compile the database. To obtain a rectangle traits-by-site matrix that can be analysed by multivariate statistics, the number of individuals possessing a particular trait category (e.g. spring breeders) was allocated to each plot, similarly to an ordinary species-by-site matrix (i.e. species were replaced by trait categories). If individuals shared more than one trait category, e.g. dimorphic species, they received an entry for each category.

### Environmental variables

In the years 1998 and 1999, dipwell and crest gauges were installed on each sampling plot to measure maximum groundwater depth (in m), mean groundwater depth (in m), duration of inundation (in weeks), and inundation height (in m). Follner and Henle (2006) correlated these plot-measurements with data from official Elbe gauges near the study site Steckby, which are daily collected by the German Waterways and Shipping Administration. By additionally accounting for evapotranspiration, a hydrological model was set up to calculate the selected hydrological variables (see Table 1) even for the year 2005, although hydrological field measurements did not continue after 1999. The reliability, the temporal and statistical robustness, as well as the application of this hydrological model was recently tested and approved in the framework of developing a bioindicator system for ecological changes in floodplains (Follner et al. 2009). Soil substrate data came also from the survey in 1999, but as the substrate type of the sampling plots did not change during the 6-year time span, I used this data for the analyses as well.

### Data analysis

Ecological studies are often biased by spatial autocorrelation, i.e. closely located samples are not independent because they can share attributes of their neighbouring samples (Dormann et al. 2007). However, independence of data points is a crucial assumption for most statistical methods. To identify spatial autocorrelation of ground beetle species richness, relative abundances and Simpson's diversity I used Moran’s I, which is a weighted correlation coefficient that detects spatial randomness or spatial clustering of variables. Values being larger than zero show positive, and values less than zero indicate negative spatial dependence of the variables. I used the knearneigh-function of the R-package spdep (Bivand 2009) using 6 plots as nearest neighbours to calculate the spatial weights matrix. Statistical significance of the autocorrelation was tested with saddlepoint approximation tests.

Principal Component Analysis (PCA) was conducted i) to identify the most important environmental variables and ii) to exclude highly correlated variables prior to further analyses. Since the environmental variables were measured on different scale units (see Table 1), I standardised them to a zero mean and unit variance to equally weight the variables. Data for substrate, management intensity, and habitat type were categorical. Therefore, these variables were transformed into dummy coded binary data before included into the analysis.

I aimed to assess the influence of environmental variables on both species assemblages and on particular species traits. A preliminary Detrended Correspondence Analysis revealed very short gradient lengths of the species and the trait datasets, suggesting low turnover rates of species and traits among the axis-gradient and thus a linear response. Therefore, I performed Redundancy Analysis (RDA) on the species (which is referred to as “species-RDA” in the following) and the traits dataset (“trait-RDA”), being much better suited for linear response patterns than unimodal models like Canonical Correspondence Analysis (Leps and Smilauer 2003). I compared the RDA models (i.e. ordination constrained by environmental variables) with unconstrained PCA models to identify the relative influence of environmental factors on the ordination models.

To determine the degree to which the occurrence of species and the occurrence of particular species traits are correlated, I performed a Procrustes rotation analysis on the species and the trait dataset. Procrustes rotation aims to find maximal congruency, i.e. similarity of data points, between two ordination models by rotating, expanding and rescaling an ordination model towards a target ordination (Legendre and Legendre 1998). To estimate if environmental variables affect the correlation I performed two Procrustes rotations: i) without environmental variables, i.e. rotation of a species-PCA model against a trait-PCA model, and ii) constrained by environmental variables, i.e. a rotation of a species-RDA model and a trait-RDA model. Statistical significance of the Procrustes rotation models were tested with a randomization test with 9,999 permutation iterations.

Variation partitioning was then used to separate the effects of different environmental compartments (predictor variables) on the variation of ground beetle life-history traits (response variable). Variation partitioning is based on RDA and tries to identify how successful a set of different predictor variables is at explaining the response variable (Legendre 2008). Hereby, the total percentage of variation explained by an RDA-model is partitioned into unique and common contributions of the predictor variables. I assumed variables related to hydrology and disturbance to explain most of the trait variation. Therefore, I divided the environmental dataset into a “hydrology” and a “disturbance” compartment (see Table 2). I additionally created a “habitat” compartment to account for the effects of environmental variables that were not measured, but being reflected in the habitat type, such as soil moisture, pH value, nutrient content etc. I assumed that species rich ground beetle assemblages should explain large parts of the trait-variation, because they should contain a large proportion of species with different biological characteristics. To account for these effects, I set up a “species diversity” compartment, containing species richness and Simpson’s diversity. Since preliminary analyses showed that soil substrate did not explain any variation in the trait-data, I excluded the soil compartment from variation partitioning.

Relative abundances of the individuals were log-transformed to reduce the skew in the data. All statistical analyses were performed with the packages vegan (version 1.15–4; Oksanen et al. 2009), spdep (version 0.4–54; Bivand9), and ade4 (version 1.4–14; Dray and Dufour 2007) in the R environment (version 2.10.0; R Development Core Team, 2009).

**Table 1.** Life-history traits of ground beetles used in this study.

*Trait*	*Trait categories*	*comments*
Body size	1 – diminutive2 – very small3 – small4 – medium	< 3.0 mm3.1 – 6.0 mm6.1 – 10.0 mm10.1 – 19 mm
Wing morphology	1 – macropterous2 – brachypterous	
Season of reproduction	1 – spring2 - autumn	From February to JuneFrom July to Oktober
Hatching season	1 – spring2 – atumnn	
Overwintering type	1 – as imago2 – as larvae	
Daily activity	1 – diurnal2 – nocturnal	
Body pubescence	1 – head2 – pronotum3 – elytra4 – hairless	
Food strategy	1 – opportunistic carnivores 2 – specialized carnivores3 – phytophagous4 – polyphagous	

## Results

Overall, 26,557 individuals from 107 species were sampled. *Agonum emarginatum* (Gyllenhal 1827; 27.7%), and *Poecilus versicolor* (Sturm 1824; 12.4%) made out 40 % of the overall individual density. 38 species were recorded with less than 5 individuals, including some stenotopic alluvial species like *Agonum dolens* (Sahlberg 1827), *Bembidion argenteolum* (Ahrens 1812) and *Omophron limbatum* (Fabricius 1776). See Appendix I for a full species list. I found only minimal spatial autocorrelation of Simpson's diversity, as seen by the relatively low Moran’s I value (M), which was only slightly greater than zero (M=0.178, p=0.015) (Table 3). Spatial dependency of both species richness (M=0.292, p=0.001) and species abundances (M=0.394, p<0.001) was little higher, nevertheless indicating a minor role of spatial autocorrelation in this study.

**Figure 2.**
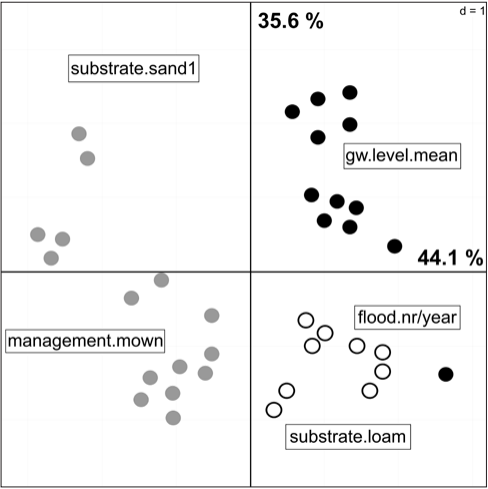
PCA of the reduced environmental dataset. Points represent the sampling plots and the colours the different habitat types: Black = floodchannels, grey = mesophilous grassland, white = humid grassland.

**Figure 3.**
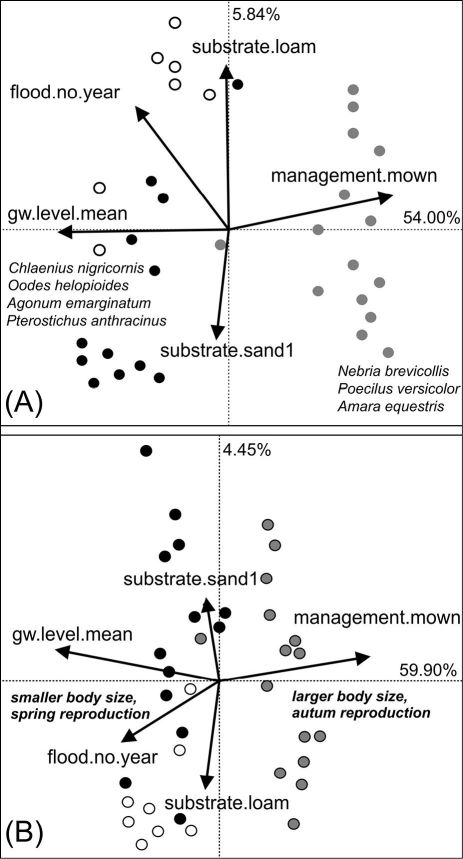
Relationship between environmental variables and species occurrence **A** and occurrence of species traits **B** by means of Redundancy Analysis. Points represent the sampling plots. Species scores omitted due to clarity. The colours indicate the habitat type of the sampling plots: black=floodchannels, grey=mesophilous grassland, white=humid grassland. Traits and species that accounted most for the explained variance along the first RDA axis are plotted in italics.

**Table 2.** Environmental variables used in the study

*Variable*	*Description*	*Data scale*	*Compartment*
Flood.height.max	Maximum flood height	Continous (cm)	Disturbance
Flood.nr/year	Number of floods per year	Continous (no.)	Disturbance
Flood.duration	Flood duration	Continous (weeks)	Disturbance
Gw.level.max	Maximum ground water depth	Continous (cm)	Hydrology
Gw.level.mean	Mean groundwater depth	Continous (cm)	Hydrology
Gw.level.varcoef	Variation coefficient of groundwater depth	Continous (no dimension)	Hydrology
Substrate.loam	Loamy substrate	Binary (0=no, 1=yes)	-
Substrate.sand1	Sandy substrate (<90% sand amount)	Binary (0=no, 1=yes)	-
Substrate.sand2	Sand (>90% sand amount)	Binary (0=no, 1=yes)	-
Substrate.silt	Silty substrate	Binary (0=no, 1=yes)	-
Management.mown	Plot mown	Binary (0=no, 1=yes)	Disturbance
Management.unused	No management	Binary (0=no, 1=yes)	Disturbance
Habitat.floodchannel	Habitat type "floodchannel"	Binary (0=no, 1=yes)	Habitat
Habitat.meadow.medium	Habitat type "mesophilous grassland"	Binary (0=no, 1=yes)	Habitat
Habitat.meadow.humid	Habitat type "humid grassland"	Binary (0=no, 1=yes)	Habitat

To reduce the complexity of the subsequent models by excluding highly correlated data, I conducted a PCA on the full environmental dataset. The full PCA model explained 68.4 % (F1: 49.4, F2: 19.3) of the total variance in the environmental data, but due to collinearity I excluded 10 environmental variables from this model (abbreviations see Table 2): gw.level.max, flood.height.max, flood.duration, gw.level.varcoef, substrate.silt, substrate.sand2, management.unused, habitat.floodchannel, habitat.meadow.medium, habitat.meadow.humid. The reduced model consisted of 5 variables and explained 79.7 % of the variation of the remaining environmental data (F1: 44.1%, F2: 35.6%). The sampling plots were ordinated along gradients of hydrological, habitat disturbance, and soil parameters (Fig. 2). Plots on the first PCA axis were mainly influenced by habitat management as well as flood and groundwater related variables, whereas soil type was the most important factor on the second axis. There are three groups of plots with similar environmental conditions, which clearly refer to the habitat types defined prior to the analyses. Habitats located in floodchannels were strongly influenced by the mean groundwater depth, whereas humid grassland habitats were more affected by the numbers of floods. The driest plots have also the highest amount of sand and are mown once or twice a year, compared to the unused floodchannels.

To evaluate how environmental variables affected the composition of species and traits I performed a Redundancy Analysis (RDA) with the reduced environmental dataset on the species and the trait dataset. The first two axes of the species-RDA explained 58.54% of the variance in the species dataset (F1: 54.00%, F2: 5.84%, Fig. 3A). It is obvious that mainly management and hydrological variables, such as the mean groundwater depth, are the main drivers affecting species occurrence. Mainly hygrophilous alluvial species, such as *Agonum* or *Bembidion* species, but also *Oodes helopioides* (Fabricius 1792) and *Pterostichus anthracinus* (Illiger 1798) are related with these environmental conditions. Therefore, plots possessing a high proportion of alluvial species were ordinated on the left side of the diagram. In contrast, the most ubiquitous species, like *Pterostichus melanarius* (Illiger 1798), *Poecilus versicolor* (Sturm 1824) and *Nebria brevicollis* (Fabricius 1792), as well as xerophilous species like *Amara equestris* (Duftschmid 1812) were rather correlated with increasing human management and higher groundwater levels and thus ordinated to the right side of the diagram. Because of the low explanatory power of the second RDA axis, soil type has only little impact on species occurrence patterns.

The first two axes of the trait-RDA explained 64.35% of the total trait variance in the dataset (F1: 59.90%, F2: 4.45%, Fig. 3B). The results indicate that especially reproductive traits and body size are strongly affected by the disturbance regime and by the hydrology of the habitats. On the left side of the ordination diagram, plots are located with a high amount of individuals reproducing in spring and hatching in summer. Most of them are additionally small sized species. On the contrary, summer/autumn breeding species and larger species are plotted more on the right side of the diagram.

Procrustes rotation analysis showed a significant correlation between species ordination and trait ordination, relatively independent from the presence of environmental constraints in the ordination (Table 4). This shows that sampling plots with a unique species composition also possess organisms with specific life-history traits. The PCA models (ordination of species and traits is not constrained by environmental variables) showed a higher congruency between each other, whereas the rotation of the RDA models tended to be less precise and showed a large part of unexplained variance, evident from the RSS values four times higher than those from the PCA model rotation.

**Figure 4.**
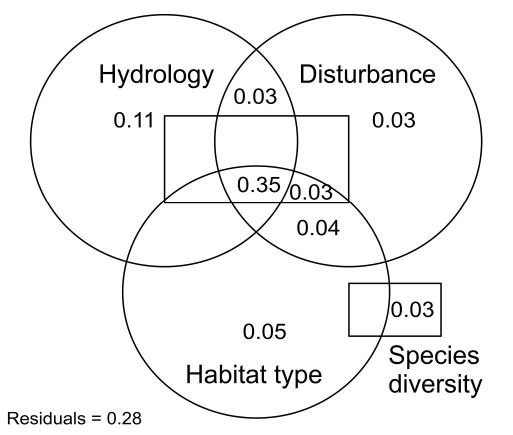
Partitioning the effects of four environmental compartments hydrology, disturbance, habitat type, and species diversity on the variation of ground beetle life-history traits. See Table 2 for a description of the variables included in each compartment. Values < 0.03 are not shown.

The environmental compartments hydrology, disturbance, habitat type, and species diversity explained 72% of the overall variation of the ground beetles life-history traits (Fig. 4). However, partitioning the effects of the predictor variables on ground beetle trait variation revealed only little explanatory power of each environmental compartment separately. Hydrology alone explained the largest part and diversity and disturbance explained the smallest part of the overall variation. The unique contribution of all compartments to the overall trait variation was 22%, whereas the common contribution (i.e. the combination of all compartments) was about 50%. In other words, the different environmental compartments explained to large degrees similar parts of the trait variation, indicating a certain amount of explanatory redundancy in the predictor variables.

## Discussion

This study tackles the problem of identifying mechanistic linkages between environmental variability, biotic characteristics of organisms and the occurrence of species in dynamic landscapes. Here I show that both species occurrence and the variation of ground beetle life-history traits are controlled by similar environmental variables. Reproductive traits and body size were found to be key traits of floodplain ground beetles enabling them to cope with management intensity and groundwater depth. Furthermore, combinations of hydrological, habitat disturbance, habitat type, and species diversity parameters, rather than their isolated effects, explained large parts of ground beetle trait variation. A main conclusion of this study is therefore that ground beetle occurrence in floodplain grasslands is mainly determined by complex interactions between environmental variability and specific life-history traits.

### Environmental variability and species occurrence

Management intensity, groundwater depth, and to a lesser degree soil substrate were the most important environmental variables driving the occurrence of species and the variation of ground beetle traits. Previous work on ground beetles in floodplains highlighted the importance of environmental variables for species occurrence in these dynamic habitats. For riverbanks, being considered as the most disturbed habitats in floodplains, Eyre et al. (2001), Kleinwaechter and Rickfelder (2007), and Framenau et al. (2002) noted that sediment type and flood disturbance are the most important factors affecting the occurrence of ground beetles. In this study I revealed that sediment type had only little influence on species occurrence and trait variation. This was not surprising, as soil dynamics, e.g. sediment erosion or deposition, are relatively low in floodplain grasslands and might therefore not be of primary importance for grassland arthropods. Rather than soil variables I found that habitat disturbance and hydrological parameters are the main factors that drive the occurrence of ground beetles in the study site. This is coincident with Antvogel and Bonn (2001), Gerisch et al. (2006) and Eyre (2006) stating that flood duration, groundwater depth and habitat management are the main factors influencing the occurrence of ground beetles in floodplains.

However, species occurrence patterns are often distance related, i.e. the values of variables (species, individuals) sampled at nearby locations are not independent from each other and lead to spatial autocorrelation (Legendre and Legendre 1998; Dormann et al. 2007). The relatively low Moran’s I values in this analysis indicate that ground beetles were rather dispersed than clustered within certain habitat types. This means that the differences in species diversity are not primarily due to spatial proximity of the sampling plots, but mainly caused by environmental variability and habitat configuration. Nevertheless, there is obviously a relationship between species assemblages located close together.

### Environmental effects on species traits

The results indicate that species assemblages of certain habitat types share unique combinations of traits, which clearly confirms the habitat templet theory. The importance of hydrological and disturbance parameters for wetland ground beetle traits is well documented in the literature. Thiele (1977) stressed the importance of floodplain species to reproduce in spring to avoid flood disturbance. Eyre et al. (2001) suggested that small body size and high mobility enable floodplain ground beetles to quickly respond to increasing disturbance. Bates et al (2006) and Lambeets et al. (2009) confirmed these assumptions, showing that several life-history traits of riverbank spiders and ground beetles are strongly affected by flood disturbance parameters. According to Ribera et al. (2001), Lambeets et al. (2009) and Sadler et al. (2006), disturbance mainly affects the dispersal capacity and the body size of ground beetles. Hence, a small body size and fully developed wings enable species to quickly evade the disturbance or quickly recolonise the disturbed plots. Overall, it is not surprising that both, the occurrence of species and their particular traits, are affected by similar environmental variables. It is suggested that only certain traits enable organisms to cope with environmental variability or extreme environmental conditions (Townsend 1997). Obviously, the set of suitable traits for coping with environmental stress is limited by nature. Therefore, “successful” strategies can be shared by several species simultaneously. The rising question of species coexistence can be best explained with functional redundancy (Petchey et al. 2007; Flynn et al. 2009) and flexible niche partitioning (Finke and Snyder 2008). Thereafter, species possessing similar life-history traits (i.e. being functionally redundant) are still able to coexist in the same habitat, because species resource use behaviour is expected to be plastic to minimise competition. Unfortunately, there are no ground beetle studies addressing functional redundancy issues, which is why an increased research on those topics is crucial to verify these assumptions.

### Combined environmental effects on species traits

Partitioning the effects of environmental variables clearly showed that a combination of all four compartments hydrology, habitat disturbance, habitat type, and species diversity explained the largest part of the overall trait variation. However, this does not automatically mean that each compartment separately is unimportant for ground beetles. In fact, each environmental compartment explained unique parts of the ground beetle trait variation, although to a comparable little amount. For example, flood disturbance is closely connected to hydrological parameters, i.e. frequently flooded plots are often the ones with the lowest groundwater depth. However, hydrological factors might not necessarily have similar impacts on the trait variation than habitat disturbance parameters. Habitat disturbance primarily affect morphological characteristics of the species, like wing morphology or body size (Ribera et al. 2001; Lambeets et al. 2009). In contrast, the alternation of hydrological parameters might more relate to reproductive traits, as shown in this study. This is also supported by Cardenas and Hidalgo (2007) noting that although most ground beetles in floodplains are spring breeders, also autumn breeding can take place at the more elevated plots. They also state that reproduction in spring might be a useful strategy for floodplain ground beetles to avoid hydrological stress for their larvae, as soil humidity in floodplains decreases considerably during the summer. I thus assume that hydrology explains mainly the variation of reproductive traits, while habitat disturbance parameters explain large parts of dispersal related traits of floodplain ground beetles. Nevertheless, the relatively high explanatory redundancy of the predictor variables suggests that there are other important variables affecting the variation of ground beetle life-history traits.

**Table 3.** Moran’s I values

*Metric*	*Moran’s I*	*p*
Species richness	0.292	0.001
Species abundances	0.394	<0.001
Simpson's diversity	0.178	0.015

**Table 4.** Procrustes rotation analysis of species and the trait dataset

	*Unconstrained(PCA)*	*Constrained by environmental dataset (RDA)*
Correlation coefficient	0.69	0.61
Residual Sum of squares	20.37	82.73
Root mean squared error	0.12	0.13
p-value	<0.001	<0.001

## Conclusions

This study confirms current knowledge about (pre-)adaptations of alluvial ground beetles to floodplain dynamics. As is evident from previous work, traits related to dispersal and reproduction are the most affected ones by flooding and are shown to change strongly with increasing inundation. This trait variation is best explained by a combination of different abiotic variables, indicating that ground beetle life-history traits are affected by multiple environmental stressors. Consequently, future ecological work and floodplain conservation measures should both focus on different facets to maintain the high trait diversity of alluvial ground beetles and the ecological functions they have in ecosystems.

Based on this work I can conclude that life-history traits can be used to predict the occurrence of organisms with certain biological characteristics to altering floodplain dynamics and to better understand ecological patterns (i.e. species occurrences). Therefore, combining traditional taxonomic approaches with current trait-based approaches is a great chance to reveal ecosystem processes and identify “rules” describing how organisms interact with their dynamic environments. Due to the high variety of different traits and strategies to cope with habitat dynamics, I appeal to intensify the application of trait-analyses also for ground beetles to increase our knowledge on processes affecting carabid-environment relationships.

## Appendix I

List of sampled individuals (). File format: Microsoft Excel Spreadsheet (xls).

Copyright notice: This dataset is made available under the Open Database License (http://opendatacommons.org/licenses/odbl/1.0/). The Open Database License (ODbL) is a license agreement intended to allow users to freely share, modify, and use this Dataset while maintaining this same freedom for others, provided that the original source and author(s) are credited.

## Appendix II

Overview of the traits included in the database and the ones used in this study (doi: 10.3897/zookeys.100.1427.app2). File format: Microsoft Word Document (doc).

Copyright notice: This dataset is made available under the Open Database License (http://opendatacommons.org/licenses/odbl/1.0//). The Open Database License (ODbL) is a license agreement intended to allow users to freely share, modify, and use this Dataset while maintaining this same freedom for others, provided that the original source and author(s) are credited.
